# Cancer risk in hospitalised asthma patients

**DOI:** 10.1038/sj.bjc.6604890

**Published:** 2009-01-27

**Authors:** J Ji, X Shu, X Li, K Sundquist, J Sundquist, K Hemminki

**Affiliations:** 1 Center for Family and Community Medicine, Karolinska Institute, Huddinge, Sweden; 2 Center for Primary Health Care Research, Lund University, Lund, Sweden; 3 Division of Molecular Genetic Epidemiology, German Cancer Research Center (DKFZ), Heidelberg, Germany

**Keywords:** asthma, cancer risk, national databases

## Abstract

Asthma is an increasingly common disorder, affecting 5–10% of the population. It involves a dysregulated immune function, which may predispose to subsequent cancer. We examined cancer risk among Swedish subjects who had hospital admission once or multiple times for asthma. An asthma research database was created by identifying asthma patients from the Swedish Hospital Discharge Register and by linking them with the Cancer Registry. A total of 140 425 patients were hospitalised for asthma during 1965–2004, of whom 7421 patients developed cancer, giving an overall standardised incidence ratio (SIR) of 1.36. A significant increase was noted for most sites, with the exception of breast and ovarian cancers and non-Hodgkin's lymphoma and myeloma. Patients with multiple hospital admissions showed a high risk, particularly for stomach (SIR 1.70) and colon (SIR 1.99) cancers. A significant decrease was noted for endometrial cancer and skin melanoma. Oesophageal and lung cancers showed high risks throughout the study period, whereas stomach cancer increased towards the end of the period. The relatively stable temporal trends suggest that the asthmatic condition rather than its medication is responsible for the observed associations.

Asthma is an obstructive lung disease in which recurrent bronchospasms cause breathing difficulties, dyspnoea and wheezing (Holgate and Polosa, 2006). The prevalence of asthma has increased during the past decades and according to a US survey, 8.9% of the adult population has ever been diagnosed with asthma (Scheller *et al*, 2006). The annual incidence rate for adults has been 2.2 cases per 1000 persons in the Nordic countries (Toren *et al*, 2004). An improved hygiene and a drop in exposure to viral and bacterial infections are thought to explain part of the increase (Maddox and Schwartz, 2002; Oh *et al*, 2004; Yeatts *et al*, 2006). An enhanced TH2 immune response followed by an increased production of cytokines of type IL-4 and IL-5 are thought to contribute to the development of asthma (Ngoc *et al*, 2005). Corticosteroids and *β*-adrenergic bronchodilators have been the principal medication for asthma, and from about 1970 these have been administered by inhalation (Chu and Drazen, 2005; Bateman *et al*, 2008). As newer agents were introduced in the 1990s, antileukotrienes targeted the bronchoconstrictor and anti-IgE block circulating IgE (Chu and Drazen, 2005). The changing asthma treatment in Sweden between 1980 and 1991 led to a 3.8-fold increase in the cost of pharmaceuticals, mainly inhaled corticosteroids, which in turn decreased the need for hospital admissions by 35% in the same period (Jacobson *et al*, 2000; Bateman *et al*, 2008).

The immune system has important functions against tumour formation (Swann and Smyth, 2007). Thus, dysregulation of the immune function in conditions such as asthma could potentially lead to cancer development. Some earlier studies have evaluated the association between a history of asthma and cancer occurrence, but the results have been inconsistent (Alderson, 1974; Vena *et al*, 1985; Markowe *et al*, 1987; Reynolds and Kaplan, 1987; Kallen *et al*, 1993; Vesterinen *et al*, 1993; Ye *et al*, 2001; Boffett *et al*, 2002; Soderberg *et al*, 2006). Many earlier studies have been small in size and short in follow-up time. To overcome these limitations, we carried out a longitudinal cohort study to quantify the subsequent cancer risk in hospitalised asthma patients, using the Swedish nationwide registers. This study is by far the largest one published on the theme, covering 140 425 patients and allowing a separate analysis of patients with multiple hospital admissions and thus with chronic asthma.

## Patients and methods

We used the Swedish Hospital Discharge Register, founded in 1964–65 by the National Board of Health and Welfare with a complete nation-wide coverage since 1987, to create a cohort of asthma patients. Only those patients with hospital admission on account of asthma were included in this study. These patients were retrieved from the registry according to the seventh (1964–68 code 241), eighth (1969–86 code 493), ninth (1987–96 code 493), and tenth (1997– code J45 and J46) International Classification of Diseases (ICD) codes. However, outpatients were not included in the Swedish Hospital Discharge Register, and therefore these patients were not included in our study. A total of 140 425 patients were identified in the registry. The study cohort was linked to the national Swedish Cancer Registry, founded in 1958 with close to 100% coverage, to ascertain all incident cancers from the start of follow-up until 31 December 2004. The Cancer Registry used a four-digit code according to ICD-7 to identify malignant tumours during the study period. Additional linkages were carried out to the national census data to obtain individual occupational status, to the National Registry of Causes of Death to identify the date of death, and to the Emigration Registry to identify the date of emigration. All linkages were carried out by the use of an individual national identification number that is assigned to each person in Sweden for his or her lifetime. This number was replaced by a serial number for each person in order to provide anonymity.

Person-years were calculated from the last hospital admission for asthma until diagnosis of cancer, death, emigration or closing date (31 December 2004), whichever came first. The follow-up time was divided into four periods: <1, 1–4, 5–9 and ⩾10 years. Standardised incidence ratios (SIRs) were calculated as the ratio of observed to expected number of cases. The expected numbers were calculated by the incidence rates for all individuals without a history of asthma, and the incidence rates in the reference group were similar to the general population in our database. The rates were standardized by 5-year age, gender, period (5-year group), socioeconomic status, and residential area (Esteve *et al*, 1994). For cancers of the female reproductive system, age at first childbirth and parity were also standardised. The 95% confidence interval of the SIR was calculated assuming a Poisson distribution, and they were rounded to the nearest two decimals (Esteve *et al*, 1994). Temporal trend was tested based on the χ^2^ distribution of the test statistic *χ*^*2*^ with 1 degree of freedom. All analyses were performed using the SAS statistical package (version 9.1; SAS Institute, Cary, NC, USA). The ethics committee at Karolinska Institute, Stockholm, Sweden, approved this study.

## Results

A total of 140 425 patients were hospitalised in Sweden for asthma during 1965–2004; among them 83 098 (59.2%) patients were hospitalised only once, 22 338 (15.9%) twice, 10 253 (7.3%) thrice, 5836 (4.2%) for four times, 3717 (2.7%) for five times, and 15 183 (8.7%) for more than five times. A total of 7421 patients developed subsequent cancer after being hospitalised for asthma, giving an overall SIR of 1.36 and a SIR of 1.24 for cancer diagnosed later than 1 year of the last hospital admission (all 1+), as shown in Table 1. Only those cancer sites with at least 60 cases during the whole follow-up period were listed. The risk for some cancer sites, such as lung cancer and leukaemia, was high during the first year, probably because of a concomitant diagnosis of asthma and cancer. Thus, we show the data for the whole follow-up period and 1+, respectively; the results agreed for significant increases and decreases for almost all the examined cancers. The highest overall increase of 2.28 was noted for lung cancer, and the increases were noted in the whole follow-up periods. Even all the other cancers, with an exception for breast and ovarian cancers and non-Hodgkin's lymphoma and myeloma, showed a significantly increased SIR. A significantly decreased SIR was noted for endometrial cancer (0.77) and skin melanoma (0.84).

Multiple hospital admissions may reflect the disease severity, and we examined cancer risk by the number of hospital admissions by starting the follow-up after the last admission (Table 2). The overall SIR was 1.23 for asthma patients who have been hospitalised once, and it was slightly increased to 1.25 for those with 2–5 hospital admissions and to 1.29 for those with more than five hospital admissions. The SIR was higher for almost all cancers when asthma patients had multiple hospital admissions, with an exception for lung, bladder and endocrine gland tumours, and leukaemia. We tested for the significance of the trend in the number of hospital admissions (see asterisk after ‘cancer site’). Stomach and colon cancers and non-Hodgkin's lymphoma showed an increasing trend with the number of admissions, whereas a reverse trend was noted for lung cancer. In those hospitalised more than five times, the SIR of kidney cancer exceeded that of lung cancer.

To study the periodic effects in respect to the changing therapeutic regimens and diagnosis criteria, we analysed cancer risk among asthma patients who were hospitalised in the 1970s, 1980s, and 1990s (Table 3). As maximally 14 years could be followed up for those who had been hospitalised in the 1990s, analyses were limited to cancers diagnosed 1–14 years after the last hospital admission. The overall SIR was marginally higher for the patients hospitalised in the 1970s compared with those hospitalised later. The highest risk (2.79) was noted for oesophageal cancer when patients were hospitalised in the 1990s. We tested for the significance of the temporal trends. The risk for stomach cancer and melanoma showed an increasing temporal trend, whereas a reverse trend was noted for non-Hodgkin's lymphoma.

## Discussion

In this population-based study, 140 425 asthma patients were identified from the Swedish Hospital Discharge Register and were followed up to 40 years, an observation time longer than that in any published study on this theme. The additional strengths of our study include its population-based prospective design and completeness of follow-up of the patients. All the data were from nation-wide databases guaranteeing reliable information. One limitation of this study is that because multiple comparisons were performed, some of the findings may be due to chance. The lack of information regarding medication and treatment and regarding the possible confounding factors, such as smoking, is another limitation. Moreover, the data from this study are not directly applicable to all patients with asthma because outpatients were not included in this study and hospitalised patients probably represent a severe and chronic clientele.

Overall, a 36% excess incidence of cancer was noted among asthma patients who had been treated in hospitals. Most earlier studies reported a reverse association or no association between asthma and the overall cancer risk (Kallen *et al*, 1993; Turner *et al*, 2005, 2006); a Swedish study from the earlier days of the Hospital Discharge Register reported a decreased mortality of 66% (Kallen *et al*, 1993). According to a recent review on cancer risk in asthma (Turner *et al*, 2006), only one Finnish cohort study has shown a positive association (Vesterinen *et al*, 1993), but significant only for men. The number of cancer cases in our study was almost the sum of cases in three earlier cohort studies (Kallen *et al*, 1993; Vesterinen *et al*, 1993; Turner *et al*, 2005). For the cancers overall, the highest risks were noted during the first year after last hospital admission for asthma, which could be due to lead time bias because of concomitant diagnosis. However, these earlier cancers were so few that the SIRs calculated for the whole follow-up period and for the whole period minus the first year were basically unchanged.

For specific cancers, some of the observed associations have been reported earlier. The increased risk for lung cancer was noted in many earlier studies (Vesterinen *et al*, 1993; Huovinen *et al*, 1997; Mayne *et al*, 1999; Boffett *et al*, 2002). Smoking is a common risk factor for lung cancer and asthma (Silverman *et al*, 2003; Eisner, 2008). The excess of bladder and kidney cancers in our study may also reflect confounding by smoking; however, the data on the number of hospital admissions and on temporal trends for kidney and lung cancers suggest that non-smoking-related factors contribute to kidney cancer, particularly towards the end of the study period. An increase in oesophageal and stomach cancers was reported earlier and partly attributed to gastro-oesophageal reflux caused by *β*-adrenergic medication (Ye *et al*, 2001). A significant increase of rectal cancer has been reported among Finnish women (Vesterinen *et al*, 1993), whereas an excess of prostate cancer has been reported in cohort studies from Japan and Australia (Ohrui *et al*, 2002; Talbot-Smith *et al*, 2003).

The increased risks in cancers of the colon, liver, and pancreas have not been reported earlier and most earlier studies have shown a reduced risk or risk close to unity (Vesterinen *et al*, 1993; Talbot-Smith *et al*, 2003; Turner *et al*, 2005). The excess of colon cancer increased with the number of hospital admissions and the SIR was 1.99 for those hospitalised more than 5 times, highest for any cancer, suggesting a true aetiological link. IL-6 trans-signalling is critically involved in the maintenance of both asthma and colon cancer (Scheller *et al*, 2006). Asthma medications may also play a role in this association (Friedman *et al*, 1998). Although a recent review reported no association with pancreatic cancer (Turner *et al*, 2006), a 56% excess was noted in our study and the risk was significant even after 1 year of hospital admission. An increased occurrence of squamous cell skin cancer has not been reported earlier. This cancer is one of the hallmarks of dysregulated immunity; another hallmark, non-Hodgkin's lymphoma, showed no increase and a decrease in those hospitalised only once. The decrease in endometrial cancer raised the possibility that dysregulated immune function among asthma patients may affect oestrogen production (Ahmed *et al*, 1999; Hemminki *et al*, 2008). The only other tumour with a decreased risk was melanoma, but only in asthmatics diagnosed in the 1970s. The increase in cervical cancer may signal vulnerability to infection by human papillomaviruses; immune impairment is a risk factor for cervical cancer. In contrast to the inverse association between asthma and leukaemia in the earlier studies, an increased risk was noted in our study. The positive associations with cancers of the upper aerodigestive tract, nervous system, and endocrine glands were confined for the first year after hospital admission, suggesting that they were due to the lead-time bias.

We examined the study group by the number of hospital admissions, using this as a surrogate for disease severity and chronicity. The overall cancer risk was not affected, but there were large differences by cancer types. The excess in stomach, colon, liver and skin cancers were most pronounced with multiple hospital admissions, and so was the protection in endometrial cancer. For oesophageal cancer, the highest risk of 2.21 was noted for those hospitalised 2–5 times. The two-fold risk of colon cancer may indicate medical surveillance.

The diagnostic criteria and treatment for asthma patients have changed during the follow-up period. We examined the temporal trends of cancer development among asthma patients. The overall risk was marginally higher for patients hospitalised in the 1970s compared with the later periods, and for the most specific cancers the risk did not change or decreased slightly during the study period. In view of the large increase in medications, the relatively constant risks observed throughout the study period suggest that the medical condition ‘asthma’ rather than its medication may be associated with cancer risk. However, the increasing temporal trend for stomach cancer calls for clinical attention.

In summary, a 36% excess incidence of cancer was noted among asthma patients. The robust relative risks and their consistency with multiple hospital admissions suggest real associations with several cancer sites and protection for endometrial cancer, more likely with the disorder than with its medication.

## Figures and Tables

**Table 1:** SIR for subsequent cancer in patients with hospitalised asthma during the follow-up time

	**Follow-up interval (years)**																	
	**<1**			**1–4**			**5–9**			**⩾10**			**All**			**All 1+**		
**Cancer site**	**O**	**SIR**	**95% CI**	**O**	**SIR**	**95% CI**	**O**	**SIR**	**95% CI**	**O**	**SIR**	**95% CI**	**O**	**SIR**	**95% CI**	**O**	**SIR**	**95% CI**
Upper aerodigestive tract	20	**1.77**	(1.08, 2.73)	37	1.11	(0.78, 1.53)	36	1.26	(0.88, 1.74)	42	1.09	(0.79, 1.48)	135	**1.21**	(1.01, 1.43)	115	1.14	(0.94, 1.37)
Oesophagus	9	1.99	(0.90, 3.79)	29	**2.14**	(1.43, 3.08)	28	**2.39**	(1.59, 3.46)	19	1.20	(0.72, 1.88)	85	**1.87**	(1.49, 2.31)	76	**1.85**	(1.46, 2.32)
Stomach	49	**2.41**	(1.78, 3.18)	98	**1.72**	(1.40, 2.10)	44	0.96	(0.70, 1.30)	68	1.20	(0.94, 1.53)	259	**1.44**	(1.27, 1.63)	210	**1.32**	(1.15, 1.51)
Colon	98	**2.34**	(1.90, 2.85)	225	**1.77**	(1.55, 2.02)	170	**1.54**	(1.32, 1.79)	210	**1.42**	(1.24, 1.63)	703	**1.65**	(1.53, 1.77)	605	**1.57**	(1.45, 1.70)
Rectum	49	**2.12**	(1.57, 2.81)	82	1.18	(0.94, 1.47)	72	1.20	(0.94, 1.51)	108	**1.33**	(1.09, 1.61)	311	**1.33**	(1.19, 1.49)	262	**1.24**	(1.10, 1.40)
Liver	48	**2.94**	(2.17, 3.90)	89	**1.88**	(1.51, 2.31)	59	**1.51**	(1.15, 1.95)	49	0.99	(0.73, 1.31)	245	**1.61**	(1.41, 1.82)	197	**1.45**	(1.25, 1.67)
Pancreas	44	**2.90**	(2.10, 3.89)	58	1.31	(1.00, 1.70)	55	**1.50**	(1.13, 1.95)	66	1.40	(1.08, 1.78)	223	**1.56**	(1.36, 1.78)	179	**1.40**	(1.20, 1.62)
Lung	285	**6.98**	(6.19, 7.84)	294	**2.41**	(2.14, 2.70)	186	**1.76**	(1.52, 2.04)	170	1.20	(1.03, 1.40)	935	**2.28**	(2.14, 2.43)	650	**1.76**	(1.63, 1.90)
Breast	71	1.09	(0.85, 1.38)	221	1.05	(0.91, 1.20)	172	0.89	(0.76, 1.04)	297	1.07	(0.95, 1.20)	761	1.02	(0.95, 1.09)	690	1.01	(0.94, 1.09)
Cervix	11	1.79	(0.89, 3.21)	30	**1.56**	(1.05, 2.23)	20	1.18	(0.72, 1.83)	32	1.28	(0.87, 1.81)	93	**1.38**	(1.11, 1.69)	82	**1.34**	(1.07, 1.66)
Endometrium	18	1.18	(0.70, 1.87)	36	0.74	(0.52, 1.02)	30	**0.68**	(0.46, 0.98)	45	0.76	(0.55, 1.01)	129	**0.77**	(0.64, 0.92)	111	**0.73**	(0.60, 0.88)
Ovary	19	1.60	(0.96, 2.50)	38	1.02	(0.72, 1.40)	29	0.89	(0.60, 1.28)	38	0.87	(0.61, 1.19)	124	0.99	(0.82, 1.18)	105	0.92	(0.76, 1.12)
Prostate	178	**2.12**	(1.82, 2.45)	346	**1.38**	(1.24, 1.53)	265	**1.19**	(1.05, 1.35)	397	**1.26**	(1.14, 1.39)	1186	**1.36**	(1.29, 1.44)	1008	**1.28**	(1.20, 1.36)
Kidney	38	**2.40**	(1.70, 3.30)	71	**1.55**	(1.21, 1.95)	60	**1.61**	(1.23, 2.07)	38	0.79	(0.56, 1.08)	207	**1.41**	(1.22, 1.61)	169	**1.29**	(1.10, 1.50)
Urinary bladder	59	**2.21**	(1.68, 2.85)	117	**1.47**	(1.21, 1.76)	87	**1.26**	(1.01, 1.56)	113	1.21	(1.00, 1.45)	376	**1.40**	(1.26, 1.55)	317	**1.31**	(1.17, 1.46)
Melanoma	15	1.02	(0.57, 1.69)	30	**0.63**	(0.43, 0.90)	42	0.93	(0.67, 1.26)	65	0.89	(0.68, 1.13)	152	**0.84**	(0.71, 0.99)	137	**0.83**	(0.69, 0.98)
Skin, squamous cell	36	**1.56**	(1.09, 2.16)	92	**1.28**	(1.03, 1.57)	83	**1.30**	(1.04, 1.61)	116	**1.32**	(1.09, 1.59)	327	**1.33**	(1.19, 1.48)	291	**1.30**	(1.16, 1.46)
Nervous system	48	**3.01**	(2.22, 3.99)	57	1.13	(0.86, 1.47)	51	1.15	(0.85, 1.51)	66	1.08	(0.84, 1.38)	222	**1.29**	(1.13, 1.48)	174	1.12	(0.96, 1.30)
Endocrine glands	28	**3.11**	(2.07, 4.51)	33	1.18	(0.81, 1.67)	29	1.19	(0.79, 1.70)	37	1.07	(0.75, 1.47)	127	**1.32**	(1.10, 1.57	99	1.14	(0.92, 1.39)
Non-Hodgkin's lymphoma	42	**1.87**	(1.35, 2.53)	54	0.78	(0.58, 1.02)	47	0.77	(0.56, 1.02)	87	1.03	(0.82, 1.27)	230	0.97	(0.85, 1.10)	188	0.87	(0.75, 1.01)
Myeloma	13	1.65	(0.87, 2.82)	24	1.02	(0.65, 1.52)	21	1.05	(0.65, 1.60)	31	1.16	(0.79, 1.65)	89	1.14	(0.91, 1.40)	76	1.08	(0.85, 1.35)
Leukaemia	40	**3.25**	(2.32, 4.42)	56	**1.51**	(1.14, 1.96)	47	**1.56**	(1.15, 2.08)	45	1.24	(0.90, 1.65)	188	**1.62**	(1.40, 1.87)	148	**1.43**	(1.21, 1.68)
All	1264	**2.40**	(2.27, 2.54)	2205	**1.38**	(1.32, 1.44)	1700	**1.21**	(1.15, 1.27)	2252	**1.16**	(1.11, 1.21)	7421	**1.36**	(1.33, 1.39)	6157	**1.24**	(1.21, 1.28)

CI=confidence interval; SIR=standardised incidence ratio.

Bold values represent 95% CI that does not include 1.00. Underlined values represent 99% CI that does not include 1.00.

**Table 2:** SIR for subsequent cancer in patients hospitalised for asthma by number of hospitalisations

	**Number of hospitalisations**								
	**1**			**2–5**			**⩾6**		
**Cancer site**	**O**	**SIR**	**95% CI**	**O**	**SIR**	**95% CI**	**O**	**SIR**	**95% CI**
Upper aerodigestive tract	65	1.15	(0.89, 1.47)	43	1.31	(0.95, 1.76)	7	0.62	(0.24, 1.28)
Oesophagus	39	**1.72**	(1.22, 2.36)	30	**2.21**	(1.49, 3.16)	7	1.45	(0.57, 3.00)
Stomach^*^	98	1.11	(0.90, 1.35)	81	**1.54**	(1.22, 1.92)	31	**1.70**	(1.15, 2.41)
Colon^**^	302	**1.43**	(1.27, 1.60)	212	**1.66**	(1.44, 1.90)	91	**1.99**	(1.60, 2.44)
Rectum	149	**1.28**	(1.08, 1.51)	78	1.12	(0.88, 1.40)	35	1.42	(0.99, 1.98)
Liver	110	**1.47**	(1.21, 1.77)	61	**1.35**	(1.03, 1.73)	26	**1.62**	(1.06, 2.38)
Pancreas	97	**1.37**	(1.11, 1.67)	59	**1.39**	(1.06, 1.80)	23	1.55	(0.98, 2.33)
Lung^**^	402	**1.96**	(1.78, 2.16)	190	**1.56**	(1.35, 1.80)	58	**1.36**	(1.04, 1.76)
Breast	399	1.04	(0.94, 1.14)	217	0.98	(0.85, 1.12)	74	0.99	(0.78, 1.24)
Cervix	46	1.30	(0.95, 1.73)	28	1.43	(0.95, 2.07)	8	1.29	(0.55, 2.55)
Endometrium	67	0.79	(0.62, 1.01)	36	**0.71**	(0.50, 0.99)	8	**0.46**	(0.19, 0.90)
Ovary	60	0.93	(0.71, 1.20)	34	0.92	(0.63, 1.28)	11	0.90	(0.44, 1.61)
Prostate	525	**1.21**	(1.11, 1.32)	359	**1.37**	(1.24, 1.52)	124	**1.33**	(1.10, 1.58)
Kidney	94	**1.27**	(1.03, 1.56)	52	1.21	(0.90, 1.59)	23	**1.59**	(1.00, 2.38)
Urinary bladder	189	**1.42**	(1.22, 1.63)	97	1.21	(0.98, 1.48)	31	1.09	(0.74, 1.55)
Melanoma	72	**0.76**	(0.59, 0.95)	48	0.90	(0.66, 1.19)	17	0.97	(0.57, 1.56)
Skin, squamous cell	151	**1.25**	(1.06, 1.47)	94	**1.26**	(1.02, 1.54)	46	**1.64**	(1.20, 2.19)
Nervous system	93	1.01	(0.81, 1.23)	58	1.19	(0.91, 1.54)	23	**1.58**	(1.00, 2.38)
Endocrine glands	70	**1.42**	(1.11, 1.79)	23	0.81	(0.51, 1.22)	6	0.64	(0.23, 1.40)
Non-Hodgkin's lymphoma	90	**0.75**	(0.60, 0.92)	71	0.01	(0.79, 1.27)	27	1.11	(0.73, 1.62)
Myeloma	42	1.08	(0.78, 1.47)	24	1.03	(0.66, 1.54)	10	1.21	(0.58, 2.24)
Leukaemia	89	**1.46**	(1.17, 1.80)	47	**1.45**	(1.07, 1.93)	12	1.18	(0.61, 2.07)
All	3407	**1.23**	(1.19, 1.27)	2028	**1.25**	(1.20, 1.30)	722	**1.29**	(1.20, 1.38)

CI=confidence interval; SIR=standardised incidence ratio.

Bold values values represent 99% CI that does not include 1.00. ^*^P-value for trend is <0.05. ^**^P-value for trend is <0.01.

**Table 3:** SIR for subsequent cancer in patients with hospitalised asthma during diagnosis period

	**1970s**			**1980s**			**1990s**		
**Cancer site**	**O**	**SIR**	**95% CI**	**O**	**SIR**	**95% CI**	**O**	**SIR**	**95% CI**
Upper aerodigestive tract	12	0.92	(0.47, 1.62)	44	1.17	(0.85, 1.57)	35	1.27	(0.88, 1.77)
Oesophagus	12	**2.73**	(1.40, 4.78)	18	1.18	(0.70, 1.87)	34	**2.79**	(1.93, 3.91)
Stomach^**^	22	0.92	(0.58, 1.39)	81	**1.36**	(1.08, 1.69)	71	**1.67**	(1.30, 2.10)
Colon	63	**1.69**	(1.30, 2.17)	216	**1.54**	(1.34, 1.75)	201	**1.67**	(1.45, 1.92)
Rectum	32	**1.51**	(1.03, 2.14)	81	1.04	(0.83, 1.30)	83	**1.29**	(1.03, 1.60)
Liver	33	**1.98**	(1.36, 2.78)	87	**1.70**	(1.36, 2.10)	54	**1.35**	(1.01, 1.76)
Pancreas	18	1.11	(0.66, 1.76)	79	**1.66**	(1.32, 2.08)	49	1.32	(0.97, 1.74)
Lung	96	**2.31**	(1.87, 2.83)	215	**1.58**	(1.38, 1.81)	238	**2.21**	(1.94, 2.51)
Breast	63	1.09	(0.84, 1.40)	239	1.01	(0.88, 1.14)	205	0.93	(0.81, 1.07)
Cervix	14	1.68	(0.92, 2.83)	29	1.39	(0.93, 2.00)	20	1.22	(0.74, 1.89)
Endometrium	8	0.64	(0.27, 1.27)	41	0.77	(0.55, 1.05)	41	0.79	(0.56, 1.07)
Ovary	16	1.23	(0.70, 2.01)	38	0.94	(0.67, 1.29)	28	0.81	(0.54, 1.17)
Prostate	97	**1.38**	(1.12, 1.68)	362	**1.22**	(1.10, 1.35)	303	**1.28**	(1.14, 1.43)
Kidney	17	1.00	(0.58, 1.61)	70	**1.46**	(1.14, 1.84)	61	**1.61**	(1.23, 2.07)
Urinary bladder	41	**1.63**	(1.17, 2.21)	115	**1.27**	(1.04, 1.52)	92	**1.29**	(1.04, 1.58)
Melanoma^*^	6	**0.39**	(0.14, 0.85)	45	0.77	(0.56, 1.03)	45	0.95	(0.69, 1.27)
Skin, squamous cell	18	1.13	(0.67, 1.78)	104	**1.25**	(1.02, 1.51)	96	**1.32**	(1.07, 1.61)
Nervous system	23	1.30	(0.82, 1.95)	75	**1.41**	(1.11, 1.76)	40	0.83	(0.59, 1.13)
Endocrine glands	14	1.49	(0.81, 2.50)	41	1.32	(0.95, 1.80)	28	1.09	(0.72, 1.58)
Non-Hodgkin's lymphoma^**^	21	1.39	(0.86, 2.13)	76	1.26	(0.99, 1.58)	45	0.85	(0.62, 1.14)
Myeloma	4	0.53	(0.14, 1.38)	31	1.19	(0.81, 1.69)	22	1.03	(0.65, 1.57)
Leukaemia	16	1.00	(0.57, 1.64)	50	0.94	(0.70, 1.24)	56	1.11	(0.84, 1.44)
All	675	**1.35**	(1.25, 1.45)	2213	**1.23**	(1.18, 1.28)	1932	**1.29**	(1.23, 1.34)

CI=confidence interval; SIR=standardised incidence ratio.

Bold values represent 95% CI that does not include 1.00. Underline values represent 99% CI that does not include 1.00. ^*^P-value for trend is <0.05. ^**^P-value for trend is <0.01.
